# Electrophorèse des protéines sériques: étude de 410 profils électrophorétiques

**DOI:** 10.11604/pamj.2019.32.161.11455

**Published:** 2019-04-08

**Authors:** Ouardia Bouayadi, Mohammed Bensalah, Nawal Rahmani, Said Assoufi, Mohammed Choukri

**Affiliations:** 1 Faculté de Médecine et de Pharmacie d’Oujda, Service du Laboratoire Central, CHU Mohammed VI d’Oujda, Maroc

**Keywords:** Electrophorèse des protéines sériques, Capillarys, profils protéiques, immunofixation, Serum protein electrophoresis, capillarys, protein profiles, immunofixation

## Abstract

L'électrophorèse des protéines sériques (EPS) est une analyse de routine dans la pratique quotidienne d'un laboratoire de biologie médicale. Le but de notre étude est d’analyser les différents profils électrophorétiques rencontrés dans notre pratique courante. Nous avons mené une étude transversale portant sur 410 échantillons sériques qui ont été collectés dans le cadre de l’analyse de routine au sein du laboratoire de biochimie du Centre Hospitalier Universitaire (CHU) Mohammed VI d’Oujda. L’électrophorèse des protéines sériques a été réalisée sur automate Capilarys2 Flex piercing de Sebia. 241 sérums de femmes et 169 sérums d’hommes ont été collectés. Âgés de 1 à 91 ans avec une moyenne d’âge de 49 ans. 19,5% des EPS étaient normales, l’hypo-albuminémie a été noté chez 34% des cas, un syndrome inflammatoire chronique dans 19,5% des cas, un syndrome néphrotique chez 2% des cas, 5,8% de nos patients avaient un bloc béta-gamma, une hypogammaglobulinémie a été observée dans 8,5% des cas, et 29 pics monoclonaux ont été notés, une bis-albuminémie a été objectivée chez 2 patients. Sur les 410 prélèvements: 92 immunofixations ont été réalisées dont 23 étaient positives (objectivant une gammapathie monoclonale). Cette étude met en évidence la variabilité des motifs de prescription d’une électrophorèse des protéines sériques, ainsi que l’importance des données cliniques pour une interprétation meilleure.

## Abstract (en)

Serum protein electrophoresis (SPE) is a routine analysis in the daily practice of a medical biology laboratory. This study aimed to analyze the different electrophoretic profiles seen in our current practice. We conducted a cross-sectional study of 410 serum samples collected during the routine analyses in the Laboratory of Biochemistry at the University Hospital Mohammed VI in Oujda. Serum protein electrophoresis was performed using automated instrument CAPILLARYS 2 FLEX-PIERCING, SEBIA. 241 sera from women and 169 sera from men were collected. Patients were aged between 1-91 years, with an average age of 49 years; 19.5% of SPEs were normal, hypoalbuminemia was found in 34% of cases, chronic inflammatory syndrome in 19.5% of cases, nephrotic syndrome in 2% of cases, 5.8% of our patients had betagamma block, hypogammaglobulinemia was found in 8.5% of cases and 29 monoclonal peaks were noted, bisalbuminemia was found in 2 patients. Out of 410 collections: 92 immunofixations were performed, of whom 23 were positive (showing monoclonal gammopathy). This study highlights the variability in prescribing serum protein electrophoresis as well as the importance of clinical data for a better interpretation.

## Introduction

L’électrophorèse des protéines sériques est une analyse de routine dans la pratique quotidienne d’un laboratoire de biologie médicale. En clinique cette analyse a été conçue principalement pour la recherche de gammapathies responsables de profils oligoclonaux, monoclonaux ou polyclonaux, mais également pour mettre en évidence un éventuel déficit en α1-antitrypsine, un syndrome néphrotique, contribuant ainsi au diagnostic de diverses pathologies et permettant ainsi leur suivi thérapeutique [1]. Cette étude propose une analyse des différents profils électrophorétiques rencontrés lors d’analyse des sérums des patients hospitalisés au sein de CHU Mohammed VI d’Oujda.

## Méthodes

Nous avons mené une étude transversale portant sur 410 échantillons sériques qui ont été collectés sur des patients hospitalisés dans le cadre de l’analyse de routine au sein du laboratoire de biochimie du CHU Mohammed VI d’Oujda. L’électrophorèse des protéines sériques a été réalisée sur des prélèvements à jeun, effectués sur des tubes secs après centrifugation. L’aspect de chaque échantillon a été noté, les échantillons hémolysés, opalescents et lactescents ont été exclus. Ces échantillons ont été stockés à +4°C puis analysés souvent dans les 2 jours suivant la réception au laboratoire (sans dépasser une semaine) par la technique d’électrophorèse capillaire sur automate Capillarys2 Flex piercing. Le dosage des protéines totales a été réalisé par la méthode de Biuret sur automate Architect Ci8200. Les immunofixations ont été réalisées sur des gels d’agarose sur Automate Hydrasis 2 Scan de Sebia.

## Résultats

**Figure 1:**
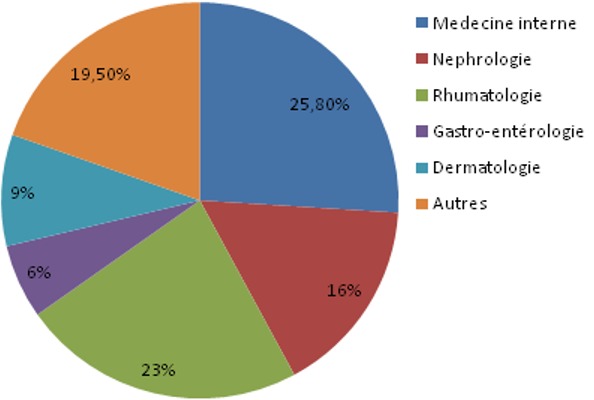
Les services prescripteurs de l’électrophorèse des protéines sériques

**Figure 2:**
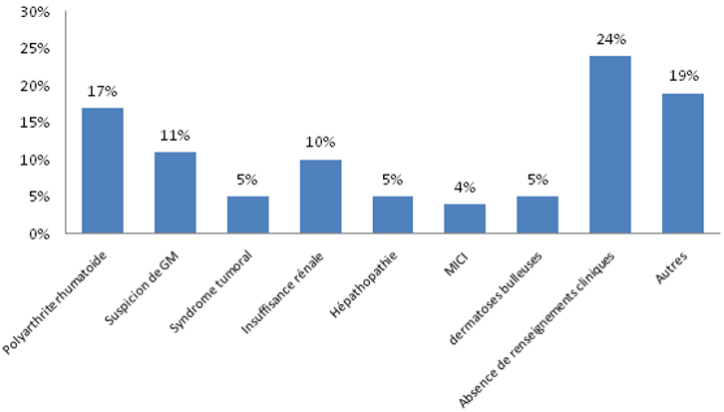
Le motif de prescription de l’électrophorèse des protéines sériques

**Figure 3:**
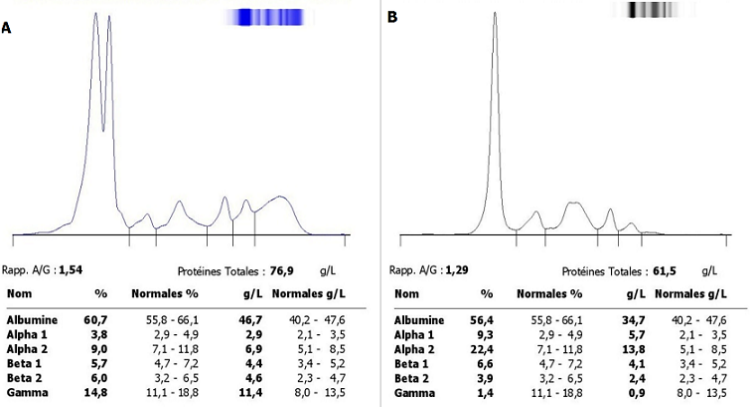
A) profil électrophorétique montrant une bis-albuminémie; B) profil électrophorétique montrant une agammaglobulinémie

Dans notre étude, 410 échantillons des différents services de CHU ont été collectés: 241 sérums de femmes et 169 sérums d’hommes. Agés de 1 an à 91 ans avec une moyenne d’âge de 49 ans. Les services les plus demandeurs étaient le Service de Médecine Interne (25,8%), la Rhumatologie (23,2%), la Néphrologie (15,8%), le Service de Dermatologie (9,2%) et la Gastro-entérologie (6,3%) (Figure 1). L’accès direct aux données des patients via le système informatique nous a permis d’exploiter les données biologiques antérieures des patients, et il a ainsi facilité l’interprétation des différents profils életrophorétiques puisque les renseignements cliniques pertinents étaient absents dans plus de 20% des cas (Figure 2). Sur les échantillons qui ont été analysés 19,5% des profils étaient qualitativement et quantitativement normales, l’hypo-albuminémie était présente dans 34% des cas; 19,5% de ces hypo-albuminémies ont accompagné un syndrome inflammatoire chronique et 2% des hypo-albuminémies étaient sévères (< 25g/l) et associés à un syndrome néphrotique, la bis-albuminémie a été objectivée chez 2 patients (Figure 3 A). Le bloc béta-gamma caractérisé par une fusion des fractions béta et gamma a été observé chez 24 patients suivis au service de médecine interne et de gastro-entérologie pour des pathologies hépatiques chroniques au stade de cirrhose.

**Figure 4:**
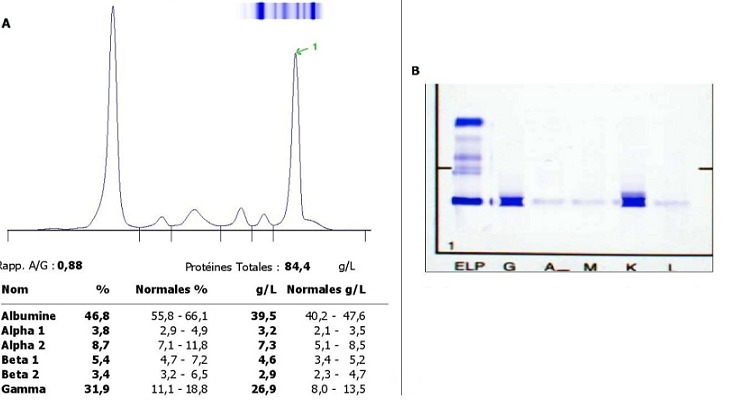
A) présence d’un pic d’allure monoclonale migrant dans la zone des gammaglobulines; B) immunofixation sérique montrant une Immunoglobuline monoclonale type IgG Kappa

L’hypo-gammaglobulinémie était présente chez 35 patients, dont 3 été associées à la présence d’une gammapathie monoclonale à chaine légère, alors que l’agammaglobulinémie n’a été objectivée que chez un enfant présentant un déficit immunitaire (Figure 3 B). Sur les 410 échantillons analysés, 29 pics d’aspect monoclonales ont été observés, 8 pics migrant dans la zone Béta, et 21 sont détectés dans la région des gammaglobulines (Figure 4 A). Par ailleurs nous n’avons pas observé de bandes monoclonales migrantes dans la zone des alpha-globulines (Tableau 1). 92 immunofixations ont été réalisées; 23 ont été trouvés à avoir une gammapathie monoclonale (Figure 4 B) (15 myélomes multiples, 8 gammapathies monoclonales de signification indéterminée (MGUS)). Le ratio hommes-femmes était de 0,9. Tous les patients avaient un âge plus de 55 ans, avec un âge plus avancé chez les femmes.

**Tableau 1:** Les résultats de l’analyse électrophorétique

Résultats de l’EPS	Pourcentage
Profil électrophorétique normal	19,5%
Hypo-albuminémie	34%
Syndrome néphrotique	2%
Bloc béta-Gamma	5,8 %
Hypo-gammaglobulinémie	8,5%
Pic d’allure monoclonal	7%
Syndrome inflammatoire chronique	19,5%
Syndrome inflammatoire aigue	7,5%
Bis-albuminémie	0,5%

## Discussion

L’électrophorèse des protéines sériques est une analyse de routine visant à rechercher les modifications qualitatives et semi-quantitatives du profil protéique. Cette analyse renseigne, entre autres, sur la présence de gammapathies monoclonales, mais également sur un déficit en α1-antitrypsine, un état inflammatoire, un syndrome néphrotique, etc. Parmi les techniques disponibles sur le marché, l’électrophorèse en gel d’agarose était encore au début des années 2000 la technique la plus utilisée, aujourd’hui fortement remplacée par la technique d’électrophorèse capillaire (EC). Cette dernière technique est l’intermédiaire entre l’électrophorèse classique de zone et la chromatographie liquide. L’EC permet une automatisation complète de l’analyse associée à une séparation en solution libre rapide et résolutive en six fractions protéiques majeures (albumine, α1-, α2-, β1-, β2-, et γ-globulines) [1].

L’analyse de l’électrophorèse des protéines sériques doit être à la fois qualitative et quantitative pour les 6 fractions protéiques. En effet, celles-ci sont évaluées de manière semi quantitative (mais avec beaucoup de précision par rapport aux techniques classiques), relativement à l’aire totale de l’ensemble du profil, et exprimées en pourcentage (%). Ces fractions protéiques relatives sont ensuite associées aux valeurs pondérales en g/L, une fois la protéinémie sérique déterminée [1]. L’électrophorèse donne, d’autre part, un reflet panoramique de l’ensemble des protéines sériques et peut à ce titre dépisté, notamment: un syndrome inflammatoire aigu: avec une hyper alpha-1 et alpha-2 liées à l’augmentation des protéines de l’inflammation; un syndrome inflammatoire chronique: avec une hyper α1 et α2 associées à l’hyper-gammaglobulinémie polyclonale liée à l’augmentation des immunoglobulines témoignant de la chronicité de la maladie; une carence martiale (augmentation de la zone bêta-1 globuline); une hémolyse intra-vasculaire (déformation de la zone alpha-2 globuline); une cirrhose (bloc bêta-gamma: fusion des fractions béta et gamma liée à l’hyper IgA polyclonal); syndrome néphrotique: hyper-alpha-2 importante liée à l’augmentation de l’a2 macroglobuline, associée à une hypo-protidémie sévère due à la fuite urinaire et à la protéinurie massive; une maladie infectieuse, auto-immune ou de système (zone alpha-1, alpha-2, bêta et surtout gamma); un déficit congénital ou acquis des immunoglobulines ou d’autres protéines (albumine, alpha-1 antitrypsine et gammaglobulines notamment) [2, 3].

Lors de l’analyse de l’EPS la plus grande importance doit être apportée à la zone des gammaglobulines qui est composée principalement d’immunoglobulines, les zones alpha-2 et béta devraient être interprétée avec prudence pour ne pas méconnaître une gammapathie monoclonale à IgA, ou une gammapathie à chaînes légères libres monoclonales qui peuvent migrer dans la zone des alpha-2 nécessitant une confirmation par immunofixation. Dans notre étude, 29 pics d’aspect monoclonales ont été trouvés dont 21(72,5%) migrant dans la zone des gammaglobulines et 8(27,5%) migrant dans la zone béta, dans l’étude de Sunita Tripathy réalisée sur une série de 150 patients suspects d’avoir un myélome multiple 87,5% des bandes monoclonales sont détectées dans la zone des gammaglobulines et seulement 12,5% des pics sont détectés dans la zone béta [4]. Dans notre étude et chez les patients ayant une gammapathie monoclonale; le sexe ratio hommes-femmes était de 0,9 et tout les patients avaient un âge plus de 55 ans.

**Figure 5:**
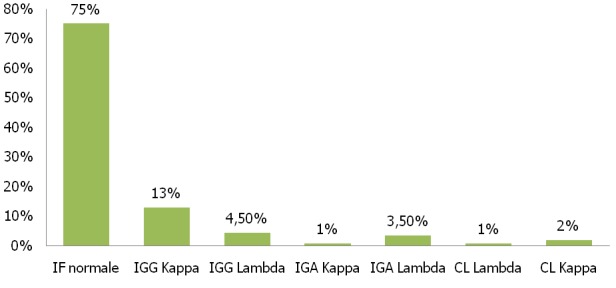
Résultats de l’immunofixation (IF)

En raison de la prescription informatisée des examens de laboratoire, l’immunofixation a été prescrite par le praticien dès le départ même en absence d’indications rationnelles et sans attendre les résultats de l’électrophorèse; dans l’objectif de diminuer la moyenne de séjour des patients en milieu hospitalier; d’où un grand nombre des immunofixations étaient revenues négatif (Figure 5). Certaines difficultés d’interprétation peuvent être rencontrées lors de l’analyse électro-phorétique; en présence d’un sérum hémolysé, la zone α2 peut être déformée en raison de la présence du complexe (haptoglobine-hémoglobine) migrant à ce niveau, et la zone β1 augmentée à cause de l’hémoglobine libre éventuellement présente. Lorsqu’il existe un reliquat de fibrinogène (analyse d’un plasma ou du sérum d’un patient sous traitement anticoagulant) un pic surnuméraire apparait au début de la zone γ, simulant un pic monoclonal. Enfin, si la concentration de la C réactive protéine est élevée (supérieure à 300 mg/L) un petit pic surnuméraire est visible dans la zone γ, en particulier s’il existe une hypogammaglobulinémie [5].

### Profils particuliers

**Figure 6:**
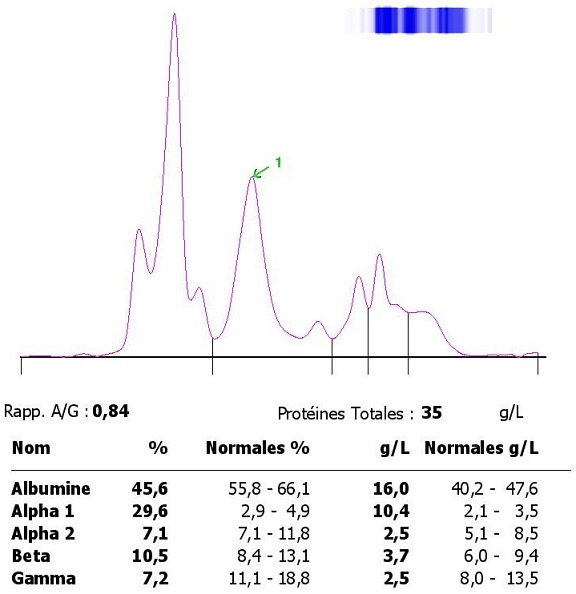
Profil électrophorétique montrant une hypoalbuminémie sévère avec des composants électrophorétiques hétérogènes d’albumine en rapport avec des fractions libres et liés d’albumine, avec un pic important migrant dans la zone α1 - α2 très compatible avec l’hypercholestérolémie et une hypogammaglobulinémie

*1^er^ cas:* il s’agit d’un patient âgé de 45 ans suivi au service de néphrologie pour un syndrome néphrotique idiopathique, patient mis sous corticothérapie, béta-lactamines, acénocoumarol et inhibiteur de l’enzyme de conversion (IEC). Son bilan biologique avait montré une fonction rénale normale avec azotémie à 0,36g/l, une créatininémie à 6,75mg/l, le bilan lipidique avait objectivé des triglycérides à 3,43g/l, un cholestérol total à 8,16 g/l, la CRP était à 55,81 mg/l. Une numération formule sanguine avait révélé: une leucocytose à 12360 /μl avec une anémie normochrome normocytaire. Le profil électrophorétique de ce patient a montré une hypo-albuminémie sévère avec des composants électrophorétiques hétérogènes d’albumine en rapport avec des fractions libres et liés d’albumine (due à une interaction des molécules de médicament avec l’albumine) ,un pic important migrant au niveau de la région alpha-1 et alpha-2 globuline (1) très compatible avec l'hypercholestérolémie et l'hypertriglycéridémie et une hypogammaglobulinémie très hétérogènes (Figure 6).

**Figure 7:**
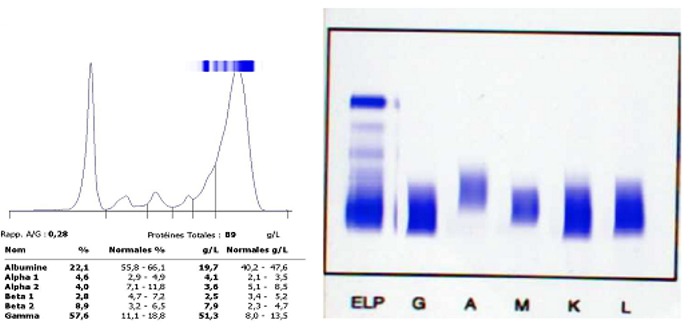
A) l’électrophorèse des protéines sériques objective un pic important d’allure polyclonal dans la zone des gammaglobulines; B) immunofixation négative avec la présence des différents types d’immunoglobulines à des taux élevés

*2^ème^ cas:* il s’agit d’une patiente âgée de 65 ans admise au service de médecine interne pour poly-adénopathies cervicales, altération de l’état général avec une asthénie. Le bilan biologique avait objectivé une anémie normochrome normocytaire agénérative avec sur frottis sanguin une plasmocytose sanguine à 35%, une CRP à 60mg/l. Le myélogramme avait montré une plasmocytose médullaire à 10% avec la présence d’un contingent de lymphocytes atypiques à 10% type: lymphoplasmocytes, lymphocytes hyperbasophiles. L’électrophorèse des protéines sériques a objectivé un pic d’allure polyclonal dans la zone des gammaglobulines, une immunofixation a été réalisée pour rejeter toute interférence avec un éventuelle monoclonalité, l’immunofixation s’est avérée négative avec la présence des différentes types d’immunoglobulines à des taux élevés en rapport avec une importante réaction immunitaire (Figure 7). Une biopsie ganglionnaire a été réalisée objectivant un lymphome malin non hodgkinien.

## Conclusion

L’électrophorèse des protéines sériques est un examen facile à réaliser peu coûteux et de grand intérêt clinique notamment pour le dépistage de gammapathies monoclonales, et la surveillance des myélomes multiples, d’autres indications sont plus subjectives dépendantes de la pratique de chaque médecin, ce qui implique la nécessité de pouvoir disposer de recommandations de bonne pratique de prescription et d’interprétation de cet examen.

### Etat des connaissances actuelles sur le sujet

- Examen de routine en biologie médicale;
- Diagnostic des gammapathies monoclonales;
- Pièges analytiques à ne pas méconnaitre.

### Contribution de notre étude à la connaissance

- Intérêt de la collaboration clinicien-biologiste pour une interprétation correcte;
- Savoir disposer de recommandations d’interprétation de l’EPS.

## Conflits d’intérêts

Les auteurs ne déclarent aucun conflit d'intérêts.
